# Towards On-Chip Self-Referenced Frequency-Comb Sources Based on Semiconductor Mode-Locked Lasers

**DOI:** 10.3390/mi10060391

**Published:** 2019-06-11

**Authors:** Marcin Malinowski, Ricardo Bustos-Ramirez, Jean-Etienne Tremblay, Guillermo F. Camacho-Gonzalez, Ming C. Wu, Peter J. Delfyett, Sasan Fathpour

**Affiliations:** 1 CREOL, The College of Optics and Photonics, University of Central Florida, Orlando, FL 32816, USA; ricardo.bustos@Knights.ucf.edu (R.B.-R.); gcamacho@Knights.ucf.edu (G.F.C.-G.); delfyett@creol.ucf.edu (P.J.D.); 2 Department of Electrical Engineering and Computer Sciences, University of California, Berkeley, CA 94720, USA; jetremblay@berkeley.edu (J.-E.T.); wu@eecs.berkeley.edu (M.C.W.); 3 Department of Electrical and Computer Engineering, University of Central Florida, Orlando, FL 32816, USA

**Keywords:** frequency combs, heterogeneous integration, second-harmonic generation, supercontinuum, integrated photonics, silicon photonics, mode-locked lasers, nonlinear optics

## Abstract

Miniaturization of frequency-comb sources could open a host of potential applications in spectroscopy, biomedical monitoring, astronomy, microwave signal generation, and distribution of precise time or frequency across networks. This review article places emphasis on an architecture with a semiconductor mode-locked laser at the heart of the system and subsequent supercontinuum generation and carrier-envelope offset detection and stabilization in nonlinear integrated optics.

## 1. Introduction

The field of integrated photonics aims at harnessing optical waves in submicron-scale devices and circuits, for applications such as transmitting information (communications) and gathering information about the environment (imaging, spectroscopy, etc.). The applications pertaining to the transmission of information include optical transceivers [1], interconnects for high-performance computing [2,3], optical switches [4], and perhaps neural networks [5]. The sensing applications can be long-range, e.g., LiDAR [6], or short-range, e.g., absorption, Raman or florescence spectroscopy [7]. This includes spectroscopy of atomic vapors [8], which is essential in realizing a miniature atomic clock. Somewhere in between these two ranges is the quest to design an on-chip frequency-stabilized comb source. The unprecedented frequency stability, coupled with the broad comb bandwidth, has had such an impact that two of its inventors, John L. Hall and Theodor W. Hänsch, are awarded the 2005 Nobel Prize in Physics for their “contributions to the development of laser-based precision spectroscopy, including the optical frequency-comb technique”.

This review paper summarizes efforts in developing an on-chip stabilized broadband supercontinuum source in the context of above sensing goals. The paper discusses in detail the applications, the physics of supercontinuum broadening and finally various integrated-photonic architectures and associated material choices. We concentrate specifically on supercontinuum from waveguides, because the attained spectrum is typically broader and flatter than the competing architecture based on microring resonators [9]. However, this comes at the cost of the need for on-chip narrow-linewidth and high-power mode-locked laser (which is yet to be demonstrated), instead of continuous-wave (CW) pump sources needed for microrings. Irrespective of the specific implementation requirements of such a supercontinuum source, some of the challenges for developing such systems are common to the whole field of integrated photonics. For example, if silicon is chosen as the primary optical material, a.k.a., silicon photonics, there exist fundamental material limitations, such as two-photon absorption and the material’s indirect bandgap. The high propagation loss of the III/V compound semiconductor competitors—which can possess direct bandgaps, hence lasing—also renders them a less than ideal alternative. Just like in optical transceivers, the solution is ushered by heterogeneous integration of various materials for different optical functionalities [10,11]. Material heterogeneity is therefore another common feature of the technologies reviewed in this paper.

## 2. Applications of Miniature Frequency Combs

**Figure 1:**
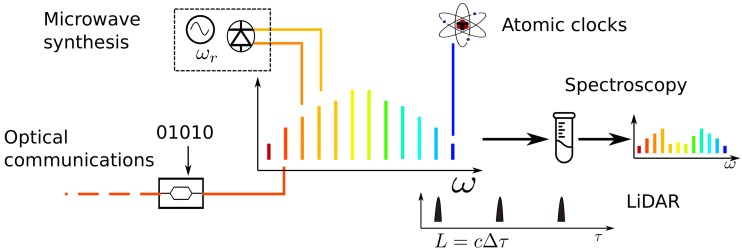
Frequency combs find applications in (i) low-noise microwave signal generation and processing, (ii) gears in atomic clocks that measure the precise frequency of atomic transitions, (iii) broadband light sources in spectroscopy. They can also be viewed as (iv) a source of densely spaced optical channels for optical communications and (v) source of high peak-power pulses in time-of-flight lidar.

The development of mode-locked laser was crucial to the proliferation of frequency combs and has spawned a multitude of applications [12], which are schematically depicted in Figure 1. Among them, spectroscopy is of prime importance. The first spectroscopic experiments concentrated on atoms from the first group of the periodic table. For instance, the Cesium D${}_{1}$ line (895 nm) was measured, thus its hyperfine constant could be calculated to high precision [13]. Further improvements were made by using self-referenced frequency combs and counter-propagating beams to avoid Doppler broadening [14]. In the initial experiments, only a single line of the frequency comb was used. However, the main advantage of the frequency comb is to use all the available frequency teeth. The next technology leap came with the invention of dual-comb spectroscopy (DCS) [15], where two locked frequency combs, with slightly detuned repetition rates, are used. In DCS, one of the combs is transmitted through the sample and then beat against the other comb producing a radio-frequency (RF) comb. The phase and amplitude information of the probing comb is mapped into the RF comb and thus the sample’s absorption spectrum can be recorded. DCS is superior—in terms of resolution, acquisition speed, accuracy and signal-to-noise ratio (SNR)—to other methods, such as high-resolution virtually imaged phased array (VIPA) disperser [16]. Since it does not require a spectrometer, i.e., a grating or an interferometer, it is a perfect candidate for an on-chip source [17]. It should be stressed that for applications in organic chemistry, it is the mid-infrared (mid-IR) region lying approximately between 4000–400 cm${}^{-1}$ or 2.5–25 μm is the most spectroscopically interesting window, and is referred to as the fingerprint region. Within this wavelength range, the rich number of rotational and vibrational excitations is sufficient for identification of organic molecules. Examples include the broadband absorption of the O-H bond of alcohols around 2.9 μm or the sharp and strong peak of the carboxyl group (C=O) near 5.5 μm [18]. Should a miniature frequency-comb spectrometer be realized in this regime, one could envision a multitude on biomedical and environmental monitoring applications. However, there are limited laser sources beyond 3 μm, hence alternative light generation methods, e.g., difference-frequency generation and optical-parametric oscillation, are required [19]. Furthermore, mid-IR detectors require cryogenic cooling, in order to limit thermal noise, and even so perform worse than their Si or InGaAs counterparts. Consequently, on-chip stabilization experiments have been primarily performed in the near-IR wavelengths, as summarized in Table 1. In this region, it is still possible to detect the overtones of vibrational resonances of certain molecules, e.g., methane (CH${}_{4}$) [20]. Additionally, the near-IR range is used in astronomical spectrograms, where frequency combs are used for calibration [21], in order to detect Doppler shifts as small as 1 cm·s${}^{-1}$. Miniaturization would enable extraterrestrial applications for frequency-comb sources.

Intimately intertwined with the topic of atomic spectroscopy is the subject of atomic clocks. Here, a narrow-linewidth laser is tuned to an atomic transition locked to one of the optical comb teeth. The frequency comb serves as the clockwork that maps the optical frequencies to microwave frequencies that can be counted by electronics. Atomic clocks form the core of international time standard disseminated globally [22]. Simultaneously, this makes them an equally capable frequency standard that would benefit from miniaturization. As the data rates and the number of data channels grow, it becomes increasingly important to synchronize the frequency among devices on the same network [23]. Miniature atomic vapors cells with vertical-cavity surface-emitting lasers (VCSELs), locked to the atomic transition, and Rubidium (Rb) vapor cells integrated with silicon waveguides [8], have already been demonstrated [23]. The next step is integration with an on-chip frequency comb source that would link the optical and microwave frequencies. Miniature atomic clocks would greatly improve the resilience of receivers for global-positioning system (GPS) against jamming [24].

Finally, there are potential applications that stem from the ability to separate individual comb lines and alter their phase and amplitude. In this manner, it is possible to synthesize arbitrary optical pulses [25] and characterize them via multiheterodyne beat [26]. Pulse shaping in the optical domain can also be used to realize programmable and tunable filters for microwave signals encoded on the optical carrier [25]. The individual comb lines can equally well-function as separate channels for coherent terabit-per-second communication [27].

## 3. Stabilization

**Figure 2:**
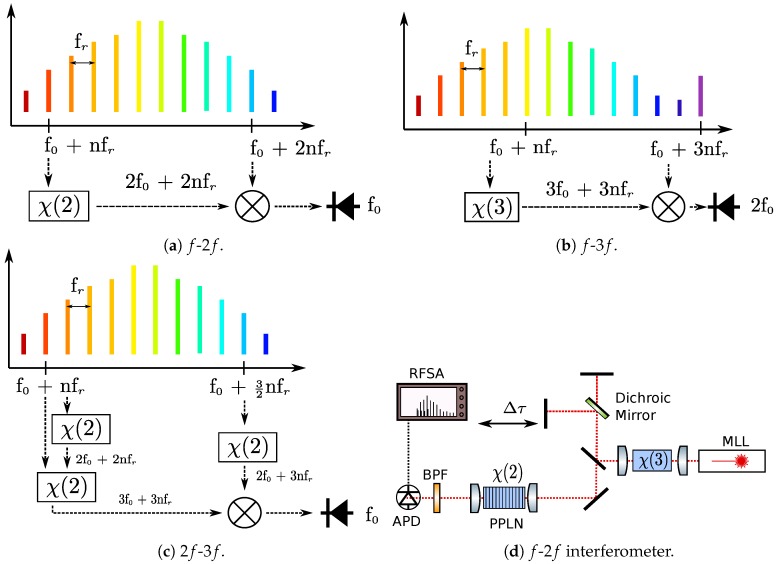
Carrier-envelope offset ($f_{0}$) detection schemes.

Frequency-comb spectra are composed of series of equally spaced lines, hence the name. The frequency comb has two free parameters, namely the repetition rate or the spacing between the comb lines, $f_{r}$, and the carrier-envelope offset (CEO) $f_{0}$, which is a measure of the phase slippage between the carrier frequency and the peak amplitude of a pulse [12], or alternatively the offset of the comb lines, with respect to zero frequency. Thus, the position of the comb lines is given by the simple relation $f_{n}=f_{0}+nf_{r}$, where *n* is an integer as depicted in Figure 2. To take full advantage of the frequency comb as an optical metrology tool, stabilizing both parameters through detection and a feedback loop back to the oscillator is demanded. Stabilization of the repetition rate is easier, as GHz-bandwidth photodetectors are readily available. Some of the repetition stabilization schemes are discussed in the Section 5. Stabilization of the CEO is more challenging, because in absence of an external reference, it is impossible to measure the optical frequency precisely. Therefore, the optical frequencies ought to be mapped into the microwave domain, so that they can be measured electronically. This is accomplished by frequency broadening of the original spectrum, frequency multiplication of one or two portions of the spectrum and subsequent measurement of the heterodyne beat between the two.

Different frequency-stabilization methods are introduced in the following. Their common challenge is that in general, frequency broadening is a non-trivial task, as the requirements for broad bandwidth and coherence must be met simultaneously. This aspect of frequency broadening is discussed in more detail in the Section 4.

As shown in Figure 2a, the most common scheme is the so-called *f*-2f self-referencing technique, where a tooth from the long-wavelength portion, $f_{0}+nf_{r}$ is frequency-doubled to $2f_{0}+2nf_{r}$ and beat against a tooth an octave apart $f_{0}+2nf_{r}$. In practice, the frequency doubling has a few nanometers of bandwidth, so actually multiple comb lines around these frequencies are used.

Another method is *f*-3f referencing, which requires two octaves of bandwidth. Here, frequency doubling is replaced with frequency-tripling, which means that the same third-order nonlinear material, i.e., with a strong χ(3) optical susceptibility, can be simultaneously used for supercontinuum generation and frequency multiplication. In this case, the beat frequency is actually $2f_{0}$, as evident in Figure 2b. But this attribution does not make a difference for the controllers used in the electronic-feedback loops, because it just affects the proportionality factor.

Finally, there are various fractional schemes, such as 2f-3f referencing that require shorter bandwidth, in this case 2/3 of an octave, but also frequency multiplication of both sides of the supercontinuum spectrum. The frequency-tripling is done in two stages, through second-harmonic and sum-frequency generations, making the whole process inefficient. The 2f-3f referencing technique is more prevalent in the case of microring resonators with limited bandwidth [28]. In one report [28], two CW lasers are locked to opposite ends of the spectrum to boost the power for 2f-3f referencing.

Several CEO detection experiments involving integrated optical waveguides are collected in Table 1. Due to the simplicity of the approach, there is particular allure of using a single, straight waveguide for both frequency doubling and supercontinuum generation. Materials such as AlN and LiNbO${}_{3}$ (LN) or even strained Si${}_{3}$N${}_{4}$, possessing both χ(3) and χ(2) nonlinear responses, can be considered. However, this comes at the price of increased power consumption for two reasons. First, efficient frequency doubling requires phase matching. In large waveguides used for supercontinuum generation, the phase-matching typically occurs for higher-order modes, as in experiments on LiNbO${}_{3}$ and AlN suggest (Table 1). This results in the CEO signal being generated from beating two different spatial modes, which limits the signal strength. Secondly, the frequency components used in *f*-2f referencing are separated by an octave, which means that they have substantially different group velocities. Therefore, as they exit the waveguide they are separated in space (or equivalently the arrival times at the detector are different). Thus, in *f*-2f referencing experiments, it is common to split the spectral components and compensate from the time delay in an interferometer, as depicted in Figure 2d. This feature is not easily available in a single waveguide with simultaneous χ(3) and χ(2) nonliterary.

Due to above-mentioned reasons, the experiment with the highest CEO SNR and lowest power consumption in Table 1 employs the architecture from Figure 2d, together with periodically poled lithium niobate (PPLN) device to achieve quasi-phase-matching to the preferred fundamental mode. To date, this has only been done in free-space optics, but progress on efforts for on-chip integration are discussed in Section 6.

Once a CEO signal with sufficient SNR (typically 25 dB) is detected, a feedback loop is used to stabilize it. In a semiconductor MLL, there are multiple factors that can affect the magnitude of the CEO. These include the gain current, operating temperature, reverse voltage of the saturable absorber and the current in an integrated phase-shifter. Furthermore, these parameters simultaneously affect the CEO and the repetition rate [34]. It is preferential to choose a free parameter and operating conditions that affect mostly the CEO, while keeping the repetition rate fixed. For semiconductor disk lasers, this has been successfully accomplished via gain current modulation [35]. Another possibility is injection locking [36], as discussed in Section 5.

Strides towards miniaturization have been made that extend beyond CEO detection. A self-referenced frequency-comb source with a fiber oscillator and silicon nitride waveguides, consuming only 5 W of electrical power, was demonstrated [37]. In this work, power-efficient repetition rate control is achieved by resistive thermal fiber heater. In another report [38], the CEO signal of a semiconductor disk laser, operating at 1.8 GHz, is stabilized via supercontinuum generation in photonic-crystal fiber. The whole system consumed 6 W of optical power. Replacing the fiber with integrated silicon nitride waveguides reduced the optical power requirement to 160 mW for a similar system, operating at 1.6 GHz [35]. However, the semiconductor disc lasers still require an external cavity with active feedback to one of the mirrors to limit amplitude and phase noise.

There also exist alternative architectures for self-referenced on-chip frequency combs. They use the spontaneous formation of solitons in microring resonators from CW background. This approach benefits from using a CW laser, instead of a mode-locked laser. An on-chip optical synthesizer has been realized based on this approach [39]. However, the spectrum of frequency combs generated in microrings follows the sech envelope function and has a limited bandwidth. Thus, from the spectroscopy viewpoint, photonic waveguides are preferable.

In the future, hybrid approaches could be possible. An example is synchronous pumping of microrings with a mode-locked laser, which should further reduce the system power consumption [40,41].

## 4. Supercontinuum Generation in Integrated Waveguides

All the self-referencing schemes in Section 3, i.e., 2f-3f, *f*-2f, and *f*-3f, require a broad-bandwidth source that is respectively 2/3 of an octave, an octave and 2 octaves wide. An octave centered around 1550 nm, corresponds to roughly 1000 nm of bandwidth. This is significantly larger than the bandwidth of typical semiconductor lasers, which is around 10 nm (see Table 2). Hence, a crucial element of any on-chip CEO detection scheme is a χ(3) waveguide, in which frequency broadening, also called supercontinuum generation, occurs. Supercontinuum generation has been thoroughly studied in the context of optical fibers [42]. Here, we review the most important findings that are relevant to obtaining a broadband, coherent supercontinuum in waveguides required for self-referencing.

The master and accurate equation describing the dynamics of fs-range pulses is

(1) $$\frac{\partial A}{\partial z}=-\frac{\alpha }{2}A+\sum_{k\geq 2}\frac{i^{k+1}}{k!}\beta_{k}\frac{\partial^{k}A}{\partial T^{k}}+i\gamma \left(1+i\tau_{\mathrm{shock}}\frac{\partial }{\partial T}\right)\left(A\left(z,t\right)\int_{-\infty }^{+\infty }R\left(T^{\prime }\right)\right|A\left(z,T-T^{\prime }{|}^{2}dT^{\prime }+i\Gamma_{R}\left(z,T\right)\right),$$

where *A* is the pulse amplitude in $W^{-1/2}$, $\beta_{i}$ are dispersion coefficients from the Taylor expansion around the center frequency, $\omega_{0}$, and γ is the nonlinear coefficient. $\tau_{\mathrm{shock}}\approx 1/\omega_{0}$ is the shock timescale, although there are more complicated expressions that account for the dispersion of the effective area [42]. R(T) is the Raman response function and various semi-empirical models are developed for it [43]. Finally, $\Gamma_{R}\left(z,T\right)$ is the stochastic noise term arising from spontaneous Raman scattering.

The dynamics of supercontinuum generation from ultrashort (100 fs range) pulse in the anomalous dispersion region, $\beta_{2}<0$, are dominated by soliton fission, as opposed to self-phase modulation in the normal dispersion case. The latter involves amplification of quantum noise via four-wave mixing (FWM) processes, which muddle the coherence of the supercontinuum. Hence, in the context of stabilization we concentrate on the anomalous dispersion regime.

Regarding the dynamics of supercontinuum generation, it is necessary to introduce the soliton number, *N*, defined as $N^{2}=\gamma P_{0}T_{0}^{2}/\left|\beta_{2}\right|$, where $P_{0}$ and $T_{0}$ refer to the peak power and duration of the input pulse in Equation (1). The first ejected soliton—which is the shortest and most energetic—has a temporal width of $T_{0}/\left(2N-1\right)$ and a peak power of ${\left(2N-1\right)}^{2}/N^{2}P_{0}$ [44]. All solitons formed during the fission process are perturbed by the higher-order dispersion terms and Raman scattering, but the perturbation of the first soliton dominates the spectrum. Under phase-matching conditions,

(2) $$D=\sum_{k\geq 2}\frac{\beta_{k}}{k!}=\frac{1}{2}{\left(2N-1\right)}^{2}\frac{|\beta_{2}|}{T_{0}^{2}},$$

the soliton can transfer energy to a linear dispersive wave [44]. To a good approximation, the right-hand side of the equation can be equated to zero. The dispersive waves are also referred to as Cherenkov radiation [45], due to similarities with radiation emitted by charged particles traveling through a dielectric medium. This equation forms the basis of dispersion engineering. Since semiconductor processing offers tight control of the waveguide geometry, it is possible to control the shape of supercontinuum spectrum in integrated waveguides (such an attribution is difficult to accomplish in largely axially symmetrical fibers). For example, it is possible to introduce a slot into the waveguide structure, effectively flattening the dispersion profile and increasing the bandwidth to two octaves [46]. This design leads the phase-matching condition from Equation (2) to be satisfied at four different wavelengths [47] with relatively flat spectrum, which would be advantageous in spectroscopic applications. Dispersion engineering is especially important in microring resonators [48], where the envelope follows the *sech*-shape of solitons. A simulated spectrum of supercontinuum generated in chalcogenide waveguides together with experimental data from [49] is appended to Figure 4b. The phase-matching condition, shown as a green curve, from Equation (2) predicts the position of dispersive waves to great accuracy.

The second nonlinear effect that perturbs the solitons is Raman scattering. In Raman scattering, some of the energy of impeding photon is transferred to electronic oscillations of molecules. This means that the soliton spectrum is continuously shifted towards red wavelengths. The speed of the frequency drift scales as $d\omega_{R}/dz∼\left|\beta_{2}\right|/T_{0}^{4}$ [50]. Importantly, spontaneous Raman scattering is an additional source of noise. It has been shown that this noise term leads to the degradation of coherence in the long-wavelength portion of the supercontinuum, hence materials with weak Raman response are preferred [31].

Apart from octave-spanning or wider bandwidth, it is necessary to ensure that the generated supercontinuum is coherent to observe the CEO beat. Coherence is defined as [42]

(3) $$\left|g_{12}^{\left(1\right)}\left(\lambda ,t_{1}-t_{2}\right)\right|=\left|\frac{\langle A_{1}^{*}\left(\lambda ,t_{1}\right)A_{2}\left(\lambda ,t_{2}\right)\rangle }{\sqrt{\langle {\left|A_{1}\left(\lambda ,t_{1}\right)\right|}^{2}\rangle \langle {\left|A_{2}\left(\lambda ,t_{2}\right)\right|}^{2}\rangle }}\right|,$$

where $A_{1}$ and $A_{2}$ refer to different pulse amplitudes in the pulse train separated by $t_{1}-t_{2}$.

Coherence requires sub-100-fs pulses [42] and the highest degree of coherence is usually observed near the soliton fission point. Also, there are several noise sources that have adverse effect on coherence. As mentioned beforehand, strong Raman effect also leads to undesired additional non-coherent photons generated through spontaneous Raman scattering. Additionally, there is quantum noise (shot noise), which is usually dominant and cannot be eradicated [51]. Furthermore, there are additional sources of noise that come from the oscillator itself, such as phase (optical linewidth), timing jitter (RF linewidth), amplitude noise and technical noise from environmental changes [52]. As the optical-fiber-based oscillators usually do not produce sufficient power for CEO detection experiments, it is necessary to amplify their output, but this leads to additional amplified spontaneous emission (ASE) noise. It has been shown that ASE noise leads to variations in the group velocity of solitons and thus timing jitter [53].

A challenge for supercontinuum generation is that as a nonlinear process, the above noise sources (ASE, Raman and shot noise) are not additive but are actually amplified through FWM processes [54]. Consequently, the FWM gain grows exponentially with pulse energy, whereas the supercontinuum bandwidth increases only linearly [55]. In other words, the minimal pulse energy that produces sufficient bandwidth would also be the point of maximum coherence.

## 5. Requirements for Semiconductor Lasers

To date, all CEO detection and stabilization experiments using integrated waveguides are performed with either fiber lasers [30], optical-parametric oscillators (OPOs) [29] or pumped vertical external-cavity surface-emitting lasers (VECSEL) [38]. Just like mode-locked fiber lasers were crucial to the development of stabilized frequency combs [13], a semiconductor laser is necessary for on-chip counterparts.

Thus, it would be prudent to compare current state-of-the art semiconductor mode-locked lasers (MLL) with these prior alternatives. As noted in reference [52], the overall frequency-comb performance is much more sensitive to the noise inside the oscillator than the noise added through subsequent amplification. The reason is that the noise inside the cavity (ASE or length variations with temperature) translates into frequency shifts or broader linewidth, whereas the same noise sources outside the cavity would increase the noise floor on the detector that is used for CEO detection.

The comparison presented here is by no means a thorough review of semiconductor MLLs and the reader is directed to references [34,56,57]. Table 2 collects performance of representative lasers. The subsequent discussion compares them against Erbium-doped fiber oscillators used in frequency-comb experiments [37,58,59,60].

With regards to the power consumption metric, nonlinear integrated waveguides typically require lower pulse energies than those in photonic-crystal fibers for supercontinuum generation. However, semiconductor-based MLL cavities are shorter and thus the repetition rates are higher than those in fiber cavities. If we take the most optimistic repetition rate value of 1 GHz from Table 2, and the best InGaP waveguides from Table 4 (which require only 2 pJ pulses, and also assume no coupling loss), the average power requirement turns out to be only 2 mW. This power is perfectly reasonable, albeit the value can quickly grow in practice. For instance, a 10-GHz InP cavity and the chalcogenide waveguides discussed later would consume 260 mW of optical power.

The next metric discussed is the pulse width. From the fiber-based comb literature [42], it is generally assumed that coherent supercontinuum generation requires 100 fs pulses, while the best MLL from Table 2 produce 900 fs pulses. However, this is not prohibitive, as it has been shown that some of the pulse compression task can be off-loaded to nonlinear waveguides. It is, for example, shown via simulations that 1 ps pulses can be compressed to 41 fs in 39-cm-long silicon nitride waveguides with ultralow propagation loss of 4.2 dB/m [65]. Similar ideas are proposed for optical fibers [66].

The subtlety lies in the fact that these two requirements are not independent. As seen in Table 2, lasers with long cavities (low repetition rates) tend to produce pulses that span tens of picoseconds. This is because, in general, longer cavities lead to larger net accumulated dispersion, which leads to wider solitons [58], i.e., $\tau_{s}=2\left|\beta_{2}\right|/\gamma P_{0}$ for $A\left(T\right)=\sqrt{P_{0}}/cosh\left(T/\tau_{s}\right)$ in the basic form of Equation (1). In this context, the path forward would probably involve further work on 1 GHz cavities with dispersion engineering or addition of gain flattering filters as in [62], in order to broaden the spectrum and reduce pulse width to picosecond level which is sufficient for further compression in nonlinear waveguides. The issues of quick gain saturation could be addressed via the breathing mode architecture [67], where the pulse is stretched before the gain section and compressed afterwards.

In the elastic-tape model of frequency combs [52,58], there exists a fixed point in the frequency spectrum, $f_{r}$, around which the comb teeth breath. In other words, the linewidth of individual comb teeth increases as we move away from $f_{r}$ [52]. In the case of active/hybrid MLLs, the fixed point is located near the carrier frequency, while in passive MLLs it is close to the zero frequency [34]. In CEO detection experiments, the comb teeth that are beat against one another (after frequency multiplication) are on the extremes of the spectrum. Hence, the linewidth of the CEO is directly proportional to the linewidth of these comb teeth. Reducing the linewidth of the CEO improves its SNR and typically a value of 25 dB is required by the locking electronics for CEO stabilization. For comparison, the free-running CEO linewidth of Erbium-doped fiber lasers is in the range of 10–1000 kHz [58]. Thus, to compare the performance of MLL in the context of frequency-comb generation, several key factors should ideally be known. They include the fixed point and its optical linewidth, as well as the spectral power density of RF phase noise, which dictates the RF linewidth and thus the magnitude of the breathing of the comb lines. However, the fixed point is rarely measured, so the optical linewidth near the carrier frequency is used as an alternative metric.

A clear distinction can be made in Table 2, between the heterogeneously integrated III-V/Si MLL and monolithic InP lasers. The former have narrower optical linewidth (e.g., 400 kHz [57]) than the latter (e.g., tens of MHz [62,64]). This is because the silicon cavities in III-V/Si have longer cavity photon lifetime than the monolithic, high-loss InP counterparts and thus wider optical linewidth through the Schawlow-Townes limit [68]. The narrowest optical linewidth of 400 kHz is still higher than typical values of tens of kHz in Erbium-doped fiber cavities, but it can be further improved with injection-locking techniques mentioned below.

The timing jitter of passive semiconductor MLLs is on the order of picoseconds, as seen in Table 2, in contrast to the femtosecond timing jitter of passive fiber cavities. However, the situation is not as dire as the numbers might imply. The 1.8 GHz VECELS used in CEO stabilization experiments [38], which are closer in repetition rate to semiconductor MLLs, have an integrated timing jitter of 60 fs (1 Hz–100 MHz) after stabilization to an external synthesizer. Therefore, in semiconductor MLLs some feedback system equalizing the repetition rate is necessary before the CEO signal can be detected.

To date, there is no single integrated passively mode-locked laser that can compete with the performance of fiber cavities simultaneously on all metrics. However, there are various methods to augment an oscillator to improve its performance. For example, hybrid-mode locking of cavity is shown to reduce the 10-dB RF linewidth from 0.9 kHz to 1 Hz [57]. In this case, hybrid-mode locking was accomplished by supplying an RF signal to the saturable absorber. We also note that the benefit of hybrid-mode locking is that it provides an access point for repetition rate control in a feedback loop in a fully self-referenced frequency-comb source.

Another possibility is optical feedback, with on-chip external cavity [61]. However, the performance of the feedback is proportional to the cavity length, as it increases the memory of the system. In one report [61], the on-chip external cavity is only twice as long as the oscillator cavity and the 3-dB RF linewidth is 6 kHz. In contrast, in a report based on a much longer (22 m) fiber-based external cavity [69], the RF linewidth is only 192 Hz. However, fabricating equally long waveguides is challenging, due to higher propagation losses of integrated photonics and limited chip space.

Another scheme is to use multitone injection locking [70]. Multitone injection locking is an extension of the injection locking technique. It reduces the optical linewidth through the use of a narrow-linewidth CW seed that is injected into the cavity [71]. The benefit of multitone injection locking is that it simultaneously reduces optical linewidth and also provides a means of controlling the repetition rate by varying the spacing of the tones. We also note that injection locking provides an alternative to the standard way of controlling $f_{0}$, via tuning the wavelength of the seed instead of modulation of the current injected into the gain section of the MLL [35].

**Figure 3:**
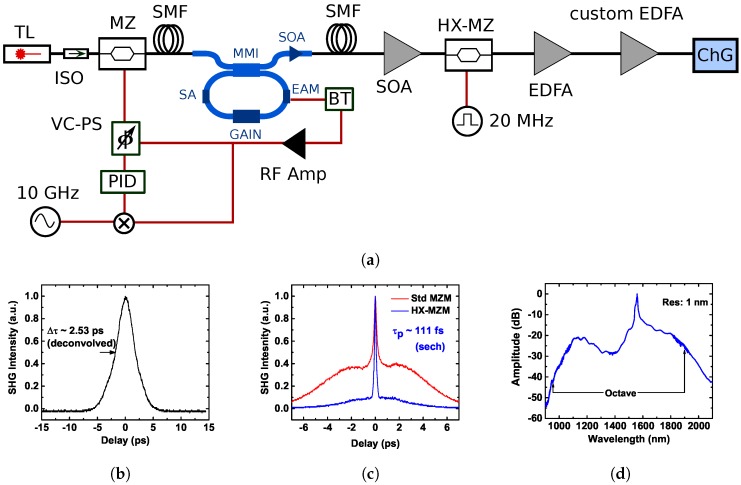
(**a**) Coupled-optoelectronic oscillator with InP MLL using multitone injection locking. The output pulses are pulse-picked, compressed and amplified to generate supercontinuum in chalcogenide (ChG) waveguides. The experimental data comes from [73]; (**b**) Autocorrelation of pulses coming out of the chip; (**c**) High-extinction modulator is essential for pulse picking, otherwise the residual pulse train is amplified leading to a wide pedestal in the autocorrelation; (**d**) Octave-spanning supercontinuum generated in ChG waveguides using the InP-based source as a seed. Abbreviations: TL—tunable laser, (HX)-MZ—(high-extinction) Mach-Zehnder modulator, MMI—multimode interferometer, EAM—electrooptic amplitude modulator, SA—saturable absorber, SOA—semiconductor optical amplifier, EDFA—Erbium-doped fiber amplifier, ISO—optical isolator, VC-PS voltage-controlled RF phase shifter, BT—bias tee, PID—proportional-integral-derivative controller, SMF—single-mode fiber.

An example of such an approach is shown in Figure 3a. The cavity used in the experiment is a 10-GHz InP colliding pulse MLL [72]. Here, the multitone injection locking is part of a coupled opto-electronic oscillator loop (COEO), i.e., the repetition rate of the MLL is detected on the electroabsorption modulator (EAM), amplified, and fed back into the Mach-Zehnder modulator, generating sidebands on the injection seed and forming a feedback loop in the system. The resonance condition is ensured by adding a phase-shifter in the loop. Another way to look at COEO is to notice that the MLL acts as a selective filter that amplifies RF modes of the cavity that overlap with the modes of the MLL. In this manner, the performance of the InP MLL is greatly improved. The free-running optical linewidth is reduced by a factor of 6000 to 100 kHz, limited by the optical linewidth of the injection seed, and the COEO loop reduces the RF linewidth by a factor of 70, yielding a 3-dB RF linewidth of 400 Hz [36]. The RF phase noise is further reduced by locking the COEO loop to an external reference, resulting in integrated timing jitter of 500 fs at 1 kHz. Unfortunately, again, the performance of COEO loop is proportional to the length of the optical delay. In this case, the optical delay line is 100 m long.

Such augmented cavity acts as a seed for supercontinuum generation in chalcogenide waveguides that are discussed in detail in Section 6. The cavity by itself produces 2.5 ps pulses (see Figure 3b) that are further amplified and compressed to 111 fs. We note that because the carrier dynamics in an EDFA is on the order of μs, every 500th pulse must be picked by a high-extinction modulator. The high-extinction modulator is essential, otherwise the residual 10 GHz pulse train is amplified, leading to a large pedestal in the autocorrelation trace as in Figure 3c.

Finally, an octave-spanning supercontinuum is achieved, as presented in Figure 3d [73]. It is anticipated that the pulse picking and multiple stages of amplification can be integrated on a single InP-based monolithic superchip.

## 6. Materials for Nonlinear Processes

As mentioned in the introduction, despite significant success of silicon photonics, some of its optical properties have certain limitations. The material has a large third-order nonlinearity, $n_{2}=\left(4.5\pm 1.5\right)\times 10^{-18}$ m${}^{2}$ W${}^{-1}$ at 1.55 μm [74], but researchers have been unable to achieve an octave-spanning supercontinuum pumped at 1.55 μm. As noted in [75], where 3/10 of an octave was demonstrated with 100 fJ pulses at 1310 nm, the two-photon and associated free-carrier absorptions clamp the maximum power inside the waveguides and hence limit the spectral broadening. Nonetheless, the pump does not have to be completely outside the two-photon absorption region supercontinuum to achieve a full octave span as in report [76]) where 1900 nm laser was used. The experimental details are provided in Table 3.

However, once appropriate on-chip sources become available, silicon is an excellent material for supercontinuum generation in the mid-IR. An octave span is already demonstrated with only 5 pJ and 300 fs pulses at 2.5 μm wavelength [79]. The bandwidth could be further improved by removing the buried oxide layer, as in suspended-membrane air-clad silicon [80], silicon-germanium [81], or silicon-on-sapphire waveguides [82].

In the nearest future, a near-IR frequency comb is the most viable path, and therefore the χ(3) limitations of silicon must be addressed by heterogeneous integration with other materials. Additionally, silicon has a centrosymmetric lattice structure, hence does not possess an intrinsic second-order nonlinear optical susceptibility, χ(2), needed for frequency doubling.

When comparing the waveguides used for CEO detection supercontinuum generation, i.e., experiments from Table 1 and Table 3, respectively, it is evident that materials with a strong χ(3) response are preferred, as it can lead to lower pulse energies required for octave-spanning supercontinuum. This is despite the fact that the Kerr nonlinearity, $n_{2}$, scales as $1/E_{g}^{4}$ with bandgap, $E_{g}$, and is inherently tied to the two-photon absorption via Kramers–Kroning relationship [83]. This means that materials with high $n_{2}$ possess a bandgap that is relatively close to the pump. For example, the transparency range of Si${}_{3}$N${}_{4}$ is 0.4–4.6 μm, whereas for silicon the transmission window is 1.2–8 μm. Due to the proximity of the bandgap, the high $n_{2}$ materials are likely to suffer from two-photon absorption. Nevertheless, within the tables, the best performing material is InGaP, which has the highest nonlinearity, despite having the highest propagation losses of 12 dB/cm [78]. Octave span is achieved with only 2 pJ of pulse energy in short (2 mm long) waveguides, which is why the high loss is not prohibitive.

The second functionality, required for the *f*-2*f* referencing scheme, is efficient SHG. For a thorough review of SHG in heterogeneous integrated photonics, the reader is directed to [84] and for a discussion of appropriate figures of merit to [85]. Table 4 collects the most important demonstrations in integrated photonics. First, we note the efficiency of SHG can be greatly improved through the power enhancement of resonant cavities such as microrings. Consequently, the highest efficiency from Table 4, as expressed in %W${}^{-1}$, is for low-loss AlN microrings, when compared to LN and GaAs waveguides, despite the AlN’s smaller χ(2) nonlinearity. However, the bandwidth of these resonant structures is limited by their quality factor, *Q*, and thus they have limited applicability to the *f*-2*f* referencing schemes, because for SHG to occur one of the comb lines would have to be tuned to the microring resonance, and tuning would amount to having an already stabilized frequency-comb source. Secondly, it is advantageous to use all comb lines that fall within the phase-matching bandwidth (usually couple of nanometers) for highest CEO SNR. Thus, in terms of architecture, straight waveguides are a better choice.

Efficient SHG requires phase matching between the pump and the signal. Since the two are separated by an octave, in terms of frequency, this leads to the phase-matching condition being satisfied for a higher-order mode at the signal wavelength as for Si${}_{3}$N${}_{4}$, GaN and AlN cases in Table 4. This is suboptimal, as it compromises the mode-overlap between the pump and signal and thus overall conversion efficiency [84], as well as the mode-overlap between the signal short-wavelength part of supercontinuum (which is usually in the fundamental mode).

As shown in Table 4, record efficiencies of 13,000% W${}^{-1}$cm${}^{-2}$ have been demonstrated in thin-film GaAs [86]. This is possible due to GaAs’s highest χ(2) response among the materials in Table 4 and a large refractive index contrast leading to a small mode area. However, this has been achieved by modal-phase matching between fundamental modes of different polarizations, i.e., the pump being in the transverse-electric (TE) and the signal in the transverse-magnetic (TM mode). This attribution has potential hurdles in *f*-2*f* referencing scheme polarization rotators must be included, because the generated SHG would be in an orthogonal polarization state to the short end of the supercontinuum.

Finally, there is the thin-film equivalent of the commercially successful PPLN technology, where the phase matching is achieved through periodic-poling of the ferroelectric LN crystals [84,90,91]. Here, efficiencies of up to 4600% W${}^{-1}$cm${}^{-2}$ have been recently demonstrated for 1550 nm pump [85] and functionality for 2 μm pump has also been shown [92,93]. Similar to the above GaAs report, operation in the fundamental mode at both pump and signal wavelengths is feasible. An advantage of the above PPLN devices is that the pump and signal can have the same TE polarization modes, hence polarization rotation is not needed.

To summarize, the most optimal on-chip CEO detection scheme, for sufficient CEO signal with the lowest pulse energy, would incorporate (a) strong χ(3) material, (b) integrated spectral splitters and delay lines, and (c) a strong χ(2) material with a device geometry leading to SHG generated in the fundamental mode and same polarization as the pump.

A sensible approach to this task is the integration of chalcogenide glass, e.g., Ge${}_{23}$Sb${}_{7}$S${}_{70}$, with thin-film LN. As discussed earlier, LN is one of the prime contenders for efficient SHG and ChG was chosen as the χ(3) material, because it is a glass that can be easily deposited on LN, without heating the substrate that would damage the thin-film LN.

Another material alternative, with strong χ(3) nonliterary, is silicon nitride. Low-loss material is typically achieved by low-pressure chemical-vapor deposition (LPCVD), which requires deposition temperature of ∼700 ${}^{{}^{\circ}}$C. Hence, in this case, it is preferred to bond LN onto patterned silicon nitride layer to avoid thermal damage to the LN thin films [94].

The fabrication flow for integration of ChG and LN has already been demonstrated together with efficient mode-conversion from the ChG layer and rib-loaded LN with only 1.5 dB loss [95]. The strongest χ(2) response of LN is along the *z* axis. Since the demonstrated thin-film PPLN devices use *y*-cut crystals, so the electrodes could be placed in the plane of the wafer, the pump field for the SHG process has to be oriented along the *z*-axis. This in turn requires the geometry of the ChG waveguide to support broadband TE supercontinuum generation, which is shown to be feasible [96].

**Figure 4:**
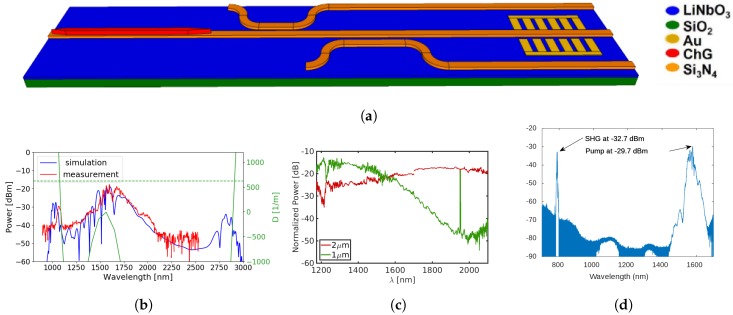
(**a**) The vision for a fully integrated *f*-2*f* referencing chip and the performance of individual components; (**b**) measured (red) octave-spanning TM supercontinuum requiring only 26 pJ of pulse energy and the simulated spectrum (blue) together with the phase-matching condition (green) that predicts the position of dispersive waves; (**c**) spectral splitters showing 30 dB extinction ratio at 2 μm; (**d**) second-harmonic generation in thin-film PPLN.

The grand vision of the final device to be demonstrated is depicted in Figure 4, together with the measured performance of discrete components. Figure 4b shows an octave-spanning TM supercontinuum generated with 240-fs-wide pulses, carrying 26 pJ of energy [49]. Figure 4c shows the performance of spectral splitters, as measured using a fiber-based supercontinuum source. Spectral separation is achieved through intermediate coupling to a higher-order mode (TE${}_{1}$), which permits definition of the whole structure in a single lithography step and avoids tiny gaps between waveguides or sharp terminations [97].

As mentioned, second harmonic was demonstrated on thin-film PPLN, reviews of which can be found elsewhere [84,85]. The first PPLN device on silicon substrates showed only 3 dB difference between the signal and the pump [90] and the recorded spectrum is shown in Figure 4d. We note that this early experiment was performed using a pulsed source and therefore there is a significant contribution from sum-frequency signal generation in the signal. Highly efficient devices, under CW pumping, were later reported [85].

**Figure 5:**
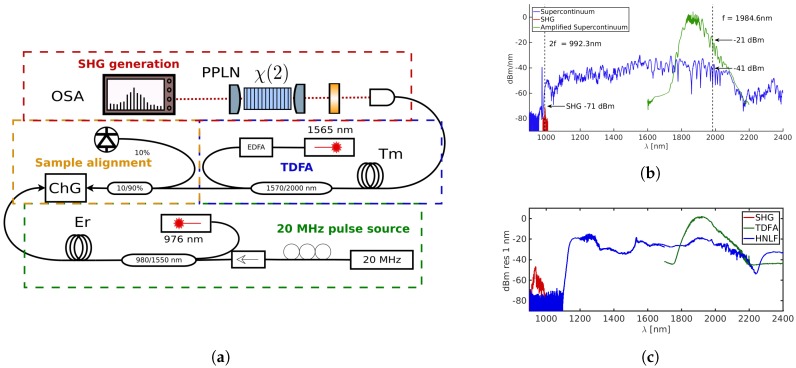
(**a**) Experimental setup for generating a second harmonic from supercontinuum, in turn, generated in chalcogenide (ChG) waveguides. A Thulium-doped amplifier (TDFA) is inserted between the ChG chip and bulk periodically poled lithium niobate (PPLN) to compensate for insertion losses. (**b**) The measured spectra in the experiment: blue—supercontinuum generated from ChG, green—amplified supercontinuum, red—second harmonic. (**c**) The same experiment performed with highly nonlinear fiber and thin-film lithium niobate.

Lastly, Figure 5 shows an experiment used to detect a second harmonic generated from supercontinuum from integrated waveguides. The long end of the supercontinuum is amplified using a custom-made Thulium-doped fiber amplifier (TDFA). The pump wavelength at 1984 nm is amplified by 20 dB to compensate for the high, 12 dB, coupling losses of the ChG chip. This is sufficient to observe a SHG signal at 992 nm at −71 dBm that falls within the short end of the supercontinuum [98]. A similar experiment with a highly nonlinear fiber and thin-film PPLN was also performed and is shown in Figure 5c. It is anticipated that integration would reduce the coupling losses rendering the TDFA unnecessary.

## 7. Future Outlook

Great progress has been made towards realizing on-chip frequency-stabilized supercontinuum-based comb sources. The integrated nonlinear components outperform the bulk counterparts, due to their smaller effective mode areas that increase the strength of interaction. The χ(3) waveguides require less power than optical fibers, in order to produce an octave-spanning supercontinuum, and in some cases the supercontinuum extends far enough to even enable *f*-3*f* referencing. Some materials, such as AlN, exhibit both χ(3) and χ(2) responses, allowing the detection of the CEO signal straight out of the waveguides. Record-high second-harmonic generation efficiencies have been demonstrated in thin-film PPLN and GaAs. The missing component is a semiconductor mode-locked laser that would serve as the heart of an on-chip frequency-comb source. In this case, heterogeneous integration of III-V gain media with long low-loss cavities, and possibly further stages of amplification and pulse compression to achieve low noise, sub-ps, 1-GHz oscillators seems to be the path forward. Subpicosecond pulses would be sufficient, provided that the nonlinear waveguides could shoulder compression to 100 fs pulses. Injection locking and hybrid-mode locking could be used to reduce the optical and RF linewidth of passive cavities. In the nearest future, an stabilized frequency combs in the near-IR is the next milestone, with potential applications in microwave synthesis, miniature atomic clocks, astronomy, and LiDAR. In the distant future, the technology could be extended to encompass the mid-IR range, where spectroscopic identification of organic molecules is possible. This is where a flat-, broadband- and stabilized-supercontinuum source would shine and open plethora of possibilities for inexpensive biomedical diagnostics and environmental monitoring.

## Figures and Tables

**Table 1:** On-chip CEO detection experiments. Abbreviations: SHG—second harmonic generation, THG—third harmonic generation, CEO SNR—carrier envelope offset signal to noise ratio.

**χ(3) Material**	**Si${}_{3}$N${}_{4}$**	**Si${}_{3}$N${}_{4}$**	**Si${}_{3}$N${}_{4}$**	**AlN**	**LiNbO${}_{3}$**
n${}_{2}$ [m${}^{2}$·W${}^{-1}$]	$2.5\times 10^{-19}$	$2.5\times 10^{-19}$	$2.5\times 10^{-19}$	$2.3\times 10^{-19}$	$1.8\times 10^{-19}$
span	600–1700 + nm	520–1700 + nm	600–1900 nm	500–4000 nm	400–2400 nm
pump wavelength	1510 nm	1550 nm	1055 nm	1550 nm	1506 nm
pulse energy	62 pJ	110 pJ	36 pJ	800 pJ	185 pJ
pulse duration	200 fs	80 fs	64 fs	80 fs	160 fs
repetition rate	80 MHz	100 MHz	1 GHz	100 MHz	80 MHz
total insertion loss	7 dB	4 dB	8 dB	8 dB	8.5 dB
χ(2) **Material**	**Strained Si**${}_{3}$N${}_{4}$	**NA**	**PPLN**	**AlN**	**LiNbO** ${}_{3}$
referencing scheme	*f*-2*f*	*f*-3*f*	*f*-2*f*	*f*-2*f*	*f*-2*f*
SHG/THG	770 nm	420 nm	680 nm	780 nm	800 nm
variable delay	No	No	Yes	No	No
CEO SNR	27 dB	23 dB	40 dB	37 dB	30 dB
reference	[29]	[30]	[31]	[32]	[33]

**Table 2:** Passively mode-locked semiconductor lasers in comparison to Erbium-doped fiber oscillators.

Material	III-V/Si	III-V/Si	III-V/Si	InP	InP	InP	Er fiber (+EDFA)
repetition rate	19 GHz	1 GHz	20 GHz	30 GHz	2.5 GHz	1 GHz	50–200 MHz
pulse duration	1.83 ps	15 ps	900 fs	900 fs	9.8 ps	70 ps	<300 fs (<100 fs)
opt. bandwidth	-	12 nm	3 nm	15 nm	3 nm	5 nm	(~50 nm)
band. threshold.	-	−10 dB	−3 dB	−10 dB	−3 dB	−10 dB	−3 dB
power	9 mW	-	1.8 mW	0.25 mW	80 uW	0.59 mW	~5 mW
	est.			coup.	coup.	coup.	(<200 mW)
3 dB optical lw	-	400 kHz	-	29 MHz	-	80 MHz	10 s kHz
RF lw	6 kHz	0.9 kHz	1.1 kHz	500 kHz	6 kHz/61 kHz	1 MHz	-
RF lw threshold	−3 dB	−10 dB	−3 dB	−20 dB	−3 dB/−20 dB	−20 dB	-
timing jitter	1.2 ps	-	-	4.5 ps	-	4.16 ps	<2 fs
int. range	0.1 MHz–	-	-	100 Hz–	10 kHz–	20 kHz–	10 kHz–
	100 MHz	-	-	30 MHz	10 MHz	80 MHz	10 MHz
reference	[61]	[57]	[56]	[62]	[63]	[64]	[37,58,59,60]

**Table 3:** On-chip supercontinuum. The $n_{2}$ for Ge${}_{23}$Sb${}_{7}$S${}_{70}$ and InGaP are inferred indirectly through measurement of γ coefficient in waveguides. For SiN, AlN, and LiNbO${}_{3}$ refer to Table 1 with CEO detection experiments.

χ(3) Material	Ge${}_{23}$Sb${}_{7}$S${}_{70}$	As${}_{2}$S${}_{3}$	InGaP	Si
$n_{2}$ [m${}^{2}$·W${}^{-1}$]	$3.7\times 10^{-18}$	$3.8\times 10^{-18}$	$2.3\times 10^{-17}$	$4.5\times 10^{-18}$
span	1030–2080 nm	1200–1700 + nm	1000–2100 nm	1150–2400 + nm
pump wavelength	1550 nm	1550 nm	1550 nm	1900 nm
pulse energy	26 pJ	60 pJ	2 pJ	4 pJ
pulse duration	240 fs	610 fs	170 fs	50 fs
repetition rate	25 MHz	10 MHz	82 MHz	200 MHz
propagation loss	0.5 dB/cm	0.6 dB/cm	12 dB/cm	1.5 dB/cm
references	[49]	[77]	[78]	[76]

**Table 4:** Second-harmonic generation in integrated waveguides.

χ(2) Material	LiNbO${}_{3}$	GaAs	AlN	GaN	Strained Si${}_{3}$N${}_{4}$
d [$\frac{1}{2}\chi \left(2\right)$]	30 pmV${}^{-1}$	119 pmV${}^{-1}$	1 pmV${}^{-1}$	8 pmV${}^{-1}$	eff. 0.02 pmV${}^{-1}$
efficiency	17%W${}^{-1}$	255% W${}^{-1}$	2300% W${}^{-1}$	0.015% W${}^{-1}$	-
efficiency	4600% W${}^{-1}$cm${}^{-2}$	13,000% W${}^{-1}$cm${}^{-2}$	-	-	-
SHG mode order	fundamental	fundamental	5th	6th	6th
architecture	waveguide	waveguide	microring	microring	microring
reference	[85]	[86]	[87]	[88]	[89]
